# Antibiotic use during radical surgery in stage I-III colorectal cancer: correlation with outcomes?

**DOI:** 10.1186/s12885-024-12550-w

**Published:** 2024-06-26

**Authors:** Mingyue Xu, Yuanyuan Chen, Panhua Li, Qianwen Ye, Shouhan Feng, Bing Yan

**Affiliations:** 1 Department of General Surgery, Hainan Hospital of PLA General Hospital, Sanya City, 572000 Hainan Province P.R. China; 2 Department of General Medicine, Hainan Hospital of PLA General Hospital, Sanya City, 572000 Hainan Province P.R. China; 3 Department of Oncology, Hainan Hospital of Chinese PLA General Hospital, No. 80 of Jianglin Road, Haitang District, Sanya City, 572000 Hainan Province P.R. China; 4 Department of Oncology, Huzhou Traditional Chinese Medicine Hospital affiliated to Zhejiang Chinese Medical University, No. 315 of South Street, Huzhou City, 313000 Zhejiang Province P.R. China

**Keywords:** Colorectal cancer, Antibiotics, Defined daily dose, Category, Gut microbiota, Survival

## Abstract

**Aims:**

Accumulating evidence indicates that the use of antibiotics (ATBs) in cancer patients is potentially correlated with patient prognosis. Interestingly, the use of these agents is not uncommon in colorectal cancer (CRC) patients during surgery; however, their prognostic value in the clinic has never been addressed.

**Materials and methods:**

Data on ATB use during surgery, including the cumulative defined daily dose (cDDD) and the number of categories, were collected. Differences in the clinical data between the low and high cDDD subgroups and between subgroups with ≤ 4 and >4 categories. Additionally, the disease-free survival (DFS) and overall survival (OS) among these subgroups and the specific categories were compared. Finally, a Cox proportional hazard model was used to validate the risk factors for the outcome.

**Results:**

The number of categories, rather than the cDDD, was a significant predictor of both DFS (*P* = 0.043) and OS (*P* = 0.039). Patients with obstruction are more likely to have a high cDDD, whereas older patients are more likely to have multiple categories. There were no significant differences in the DFS (log rank = 1.36, *P* = 0.244) or OS (log rank = 0.40, *P* = 0.528) between patients in the low- and high-cDDD subgroups, whereas patients with ≤ 4 categories had superior DFS (log rank = 9.92, *P* = 0.002) and OS (log rank = 8.30, *P* = 0.004) compared with those with >4 categories. Specifically, the use of *quinolones* was harmful to survival (DFS: log rank = 3.67, *P* = 0.055; OS: log rank = 5.10, *P* = 0.024), whereas the use of *macrolides* was beneficial to survival (DFS: log rank = 12.26, *P* < 0.001; OS: log rank = 9.77, *P* = 0.002). Finally, the number of categories was identified as an independent risk factor for both DFS (HR = 2.05, 95% CI: 1.35–3.11, *P* = 0.001) and OS (HR = 1.82, 95% CI: 1.14–2.90, *P* = 0.012).

**Conclusions:**

The cDDD of ATBs during surgery in stage I-III CRC patients did not correlate with outcome; however, patients in multiple categories or a specific category are likely to have inferior survival. These results suggest that particular caution should be taken when selecting ATBs for these patients in the clinic.

## Introduction

Colorectal cancer (CRC) remains a life-threatening disease [1, 2], with approximately 592,232 newly diagnosed individuals and 309,114 new deaths in 2022 in China [1].

Fortunately, many noninvasive approaches, such as fecal miRNA signature and fecal immunochemical tests are well-developed and could contribute to early diagnosis of the disease [3, 4]. Furthermore, owing to these technological advances, it is foreseeable that an increasing number of patients could be cured by radical resection at early stages in the future.

Postoperative infections are reportedly still unsolved complications in CRC patients, with an incidence ranging from 15 to 35% [5]. Notably, these infections can not only prolong the length of hospitalization, but also significantly reduce overall survival (OS) [5]. Many previous investigations have attempted different approaches involving the prophylactic use of antibiotics (ATBs) to reduce these infections [5–9]; however, in recent years, accumulating evidence has indicated that the prophylactic use of ATBs could result in inferior survival. For example, Derosa et al. included 121 advanced renal cell carcinoma (RCC) and 239 non-small cell lung cancer (NSCLC) patients who received immunotherapy with or without ATBs (mainly *β-lactam* or *quinolones*) concurrently, and their results indicated that patients with ATB use was associated with significantly worse progression free survival (PFS) and OS [10]. Huang et al. conducted a pooled analysis of 2740 various advanced cancer patients who received such therapies and found that ATB use was strongly correlated with poor PFS and OS [11]. In addition, ATB use was found to be a negative indicator in patients with other malignancies who accepted targeted therapy [12] or chemotherapy [13]. In CRC, exposure to ATBs has also been reported to be negatively linked to poor survival in metastatic patients treated with bevacizumab [14] or 5-Fu based chemotherapy [15]; however, another study indicated that ATB use could improve the efficacy of oxaliplatin-based rather than irinotecan-based chemotherapy in advanced settings [16]. Notably, these studies studied only the “exposure” or the “use” of ATBs, and less attention has been given to the cumulative dosage or category of ATBs. Tinsley et al. studied 291 advanced cancer patients and reported that those who received multiple courses or prolonged ATB treatment (equal to a high cumulative dosage) may have poor PFS and OS [17]. In addition, Geum et al. further indicated that broad-spectrum ATBs, such as *piperacillin*/*tazobactam* significantly reduced PFS in NSCLC patients who received nivolumab (an agent for immunotherapy) [18]. Nonetheless, there are no reports on the influence of the cumulative dosage or category during radical resection on the survival of CRC patients.

Based on this background, we aimed to determine the prognostic value of ATB use (including the cumulative defined daily dose [cDDD] and the number of categories) during radical resection in stage I-III CRC patients.

## Methods

### Patients

Data from patients with colorectal adenocarcinoma who underwent radical resection between December 2012 and December 2021 at Hainan Hospital of PLA General Hospital were retrospectively collected. Clinical features were retrieved from the archived medical records including age (≤ 50 years [y] vs. >50 y [19]), gender (female vs. male), type of resection (laparoscope vs. laparotomy), obstruction (yes vs. no) and carcinoembryonic antigen (CEA) level (normal vs. elevated vs. unknown). The pathological TNM stage was classified by postoperative report according to the eighth version of the AJCC manual. Patients with or without tumor deposits were also listed as having no specific consideration of the quantities. Patients were not included if they: had received any preoperative anticancer therapies, had a history of other malignancies, had suspected suspect distant lesion(s), had a history of ATB use before the occurrence of symptoms, or refused follow-up or were lost to follow-up. Our study was approved by the ethics committee of Hainan Hospital of PLA General Hospital (ID: S2023-12), and the requirement for written or oral informed consent was waived by the committee.

### Collecting the ATB data and calculating the cDDD

All the ATB data collected during surgery (mainly 24–48 h before surgery and before discharge for patients without emergency complications) were collected. The cDDD was calculated by the cumulative dosage multiplied by the defined daily dose, the conception of which was defined by the WHO (http://atcddd.fhi.no/ddd/definition_and_general_considera/) and then divided by the length of hospitalization (days).

### Statistical analysis

Disease-free survival (DFS) and OS are the primary endpoints [20]. Patients were classified into subgroups of low cDDD (< 7.72) vs. high cDDD (≥ 7.72) and ≤ 4 categories vs. >4 categories subgroups. These cutoff points were chosen based on the median of the data since neither of them exhibited a Gaussian distribution, as tested by the Kolmogorov-Smirnov test (cDDD: Z = 1.74, *P* = 0.005; number of categories: Z = 4.24, *P* < 0.001). Clinical outcome differences were compared between the low- vs. high-cDDD subgroups and the ≤ 4 categories vs. >4 categories subgroups using the chi-square test or Fisher’s exact test. DFS and OS were estimated using Kaplan-Meier (K-M) curves, and differences according to cDDD and the number of categories were tested using the log-rank test. A Cox proportional hazards model was fit to identify potential risk factors for DFS and OS. Finally, subgroups according to cDDD or the number of categories were tested as predictors of DFS and OS using receiver operating characteristic (ROC) curve analysis. All analyses were conducted using SPSS 20.0 (SPSS Inc., Chicago, IL, USA). All hypothesis tests were two-sided. An alpha level of 0.05 was used to determine statistical significance.

## Results

### Basic characteristics of the cohort

**Fig. 1:**
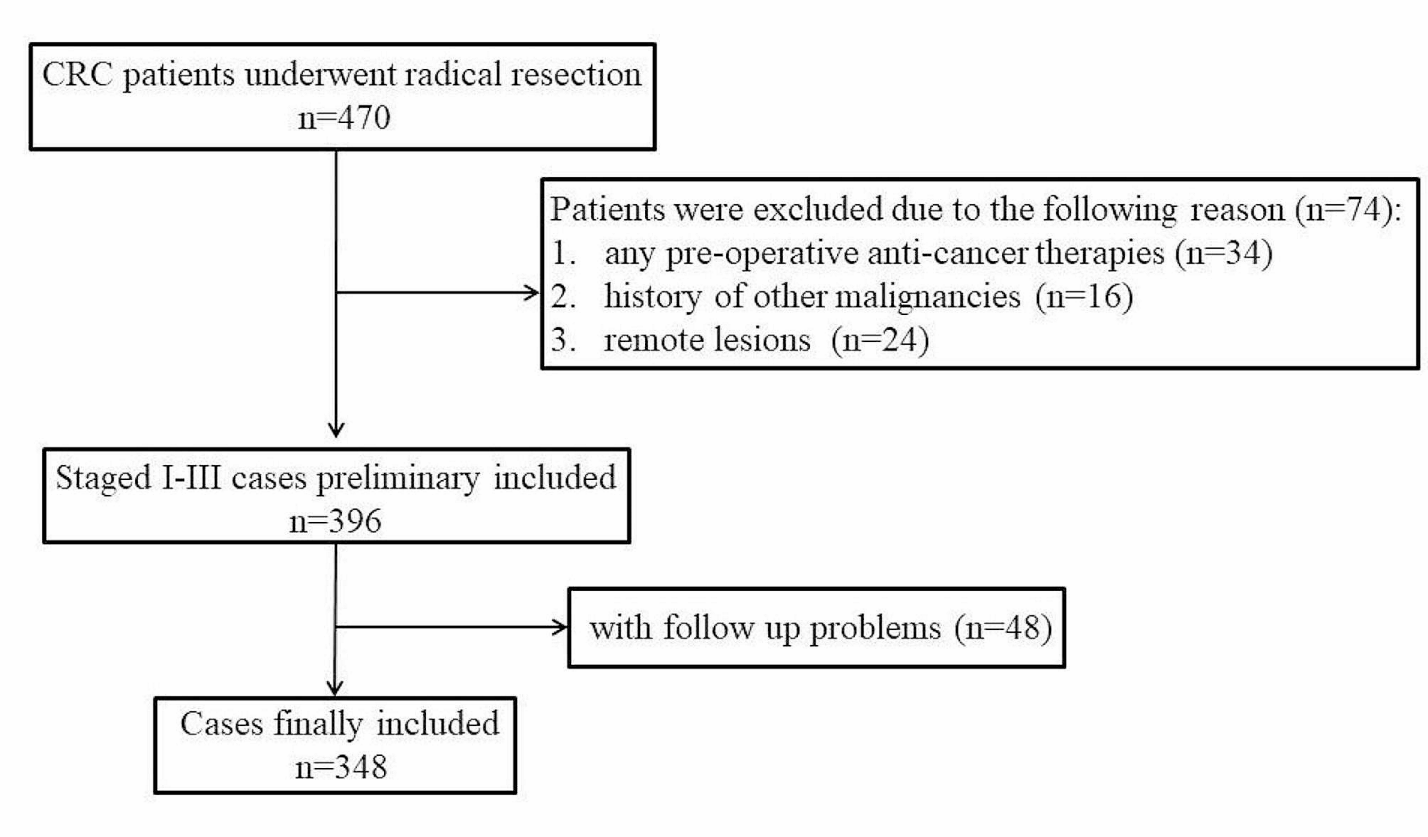
Patient inclusion procedure CRC: colorectal cancer

**Fig. 2:**
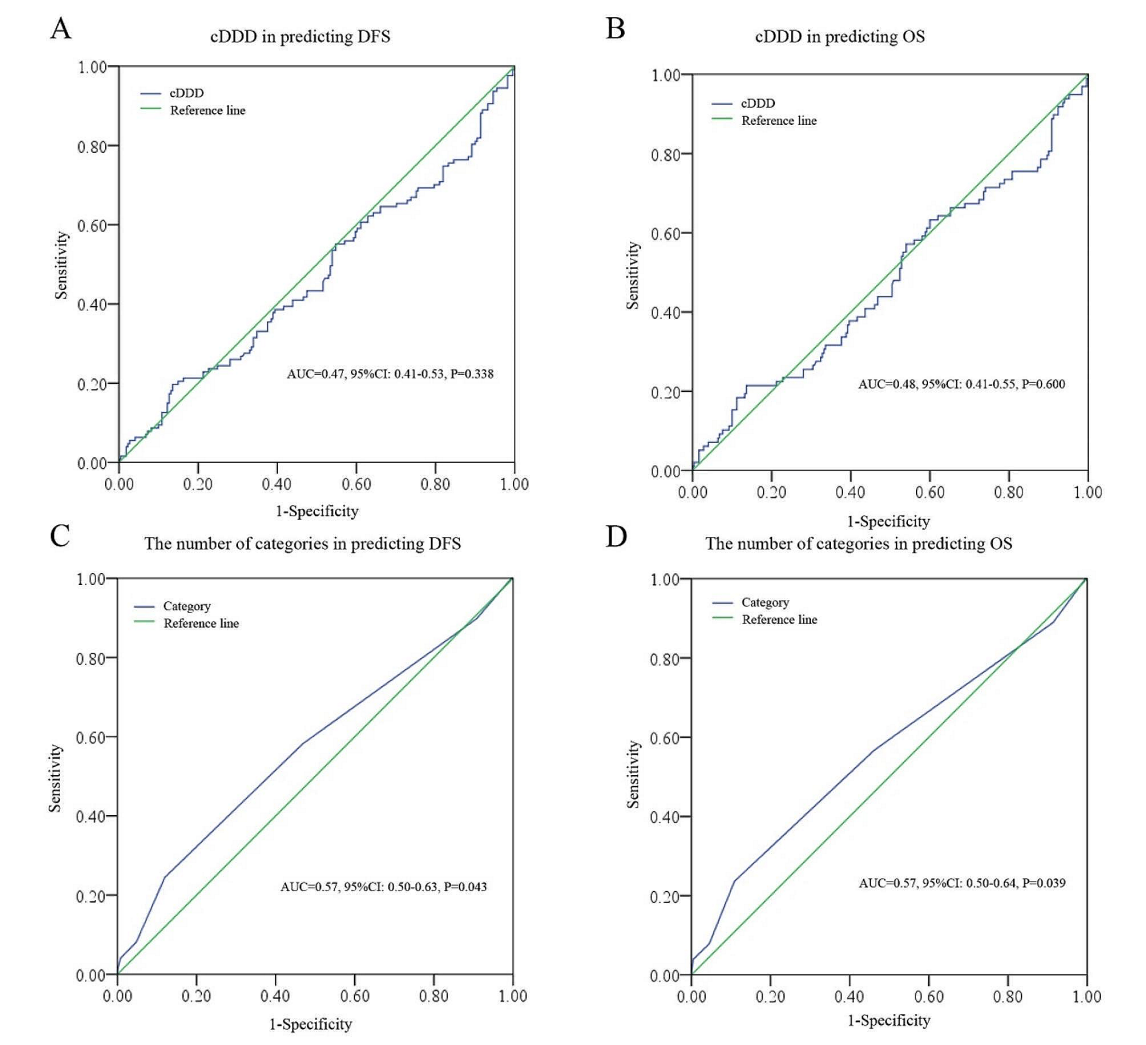
ROC analysis of cDDD (**A**, **B**) and the number of categories (**C**, **D**) in predicting DFS and OS

As shown in Fig. 1, a total of 348 patients were included in the final cohort. The median age of the patients was 62 years (y) (range: 21–90 y) and the median follow-up was 58 months (m) (range: 1–134 m). The median cDDD was 7.72 and the median number of categories was 3.5 (4 was then taken as the cut-off point). The categories of ATBs were as follows: one (*n* = 1); two (*n* = 32); three (*n* = 141); four or more (*n* = 174). The specific categories: *β-lactams* (yes vs. no: 345 vs. 3), *quinolones* (yes vs.no: 41 vs. 307), *macrolides* (yes vs. no: 296 vs. 52), *nitroimidazoles* (yes vs. no: 344 vs. 4); others categories, including *aminoglycosides*, *glycopeptides*, and *antifungals* were rare. Based on the ROC analysis, only the number of categories was significant in predicting DFS (*P* = 0.043) and OS (*P* = 0.039), whereas the cDDD was not significant in predicting DFS (*P* = 0.338) or OS (*P* = 0.600) (Fig. 2).

### Clinical data differences among the low- vs. high-cDDD subgroups and the ≤ 4 categories vs. >4 categories subgroups

As shown in Table 1, patients with obstruction were more likely to have a high cDDD (*P*<0.001), whereas older patients were more likely to have multiple categories (*P* = 0.047). No other differences were found in the other clinical data among these subgroups.

**Table 1:** Clinical data distribution differences in different cDDD or number of categories subgroups

		cDDD			The number of categories		
	*N*.	Low (*n*/%)	High (*n*/%)	*P*	≤ 4 (*n*/%)	>4 (*n*/%)	*P*
**Age (y)**				0.434^#^			0.047^#*^
≤ 50	75	41 (54.67)	34 (45.33)		69 (92.00)	6 (8.00)	
>50	273	133 (48.72)	140 (51.28)		225 (82.42)	48 (17.58)	
**Sex**				0.911^#^			0.123^#^
Female	124	61 (49.19)	63 (50.81)		110 (88.71)	14 (11.29)	
Male	224	113 (50.45)	111 (49.55)		184 (82.14)	40 (17.86)	
**Resection**				0.073^#^			0.147^#^
Laparoscope	295	154 (52.20)	141 (47.80)		253 (85.76)	42 (14.24)	
Laparotomy	53	20 (37.74)	33 (62.26)		41 (77.36)	12 (22.64)	
**Obstruction**				<0.001^#*^			0.392^#^
Yes	47	11 (23.40)	36 (76.60)		37 (78.72)	10 (21.28)	
No	301	163 (54.15)	138 (45.85)		257 (85.38)	44 (14.62)	
**Tumor location**				0.068^#^			0.316^#^
Right	92	38 (41.30)	54 (58.70)		81 (88.04)	11 (11.96)	
Left	256	136 (53.12)	120 (46.88)		213 (83.20)	43 (16.80)	
**Histological differentiation**				0.758^#^			0.837^#^
Well + moderate	299	151 (50.50)	148 (49.50)		252 (84.28)	47 (15.72)	
Poor	49	23 (46.94)	26 (53.06)		42 (85.71)	7 (14.29)	
**CEA level**				0.586^#^			0.423^&^
Normal	198	99 (50.00)	99 (50.00)		167 (84.34)	31 (15.66)	
Elevated	121	58 (47.93)	63 (52.07)		100 (82.64)	21 (17.36)	
Unknown	29	17 (58.62)	12 (41.38)		27 (93.10)	2 (6.90)	
**Deposits**				0.641^#^			0.669^#^
Yes	48	26 (54.17)	22 (45.83)		42 (87.50)	6 (12.50)	
No	300	148 (49.33)	152 (50.67)		252 (84.00)	48 (16.00)	
**T stages**				1.000^#^			0.594^#^
T_1_ + T_2_	77	39 (50.65)	38 (49.35)		67 (87.01)	10 (12.98)	
T_3+_T_4_	271	135 (49.82)	136 (50.18)		227 (83.76)	44 (16.24)	
**N stages**				0.666^#^			0.298^#^
N_0_	195	95 (48.72)	100 (51.28)		161 (82.56)	34 (17.44)	
N_1+_N_2_	153	79 (51.63)	74 (48.37)		133 (86.93)	20 (13.07)	
**TNM stage**				0.824^#^			0.499^#^
I	57	29 (50.88)	28 (49.12)		48 (84.21)	9 (15.79)	
II	138	66 (47.83)	72 (52.17)		113 (81.88)	25 (18.12)	
III	153	79 (51.63)	74 (48.37)		133 (86.93)	20 (13.07)	

^#^based on Chi-Square test; ^&^based on Fisher’s exact test; ^*^with significant statistical difference

cDDD: cumulative defined daily dose; CEA: carcinoembryonic antigen; TNM: tumor-node-metastasis; N: number

### Survival differences among the low- vs. high-cDDD subgroups and the ≤ 4 categories vs. >4 categories subgroups

**Fig. 3:**
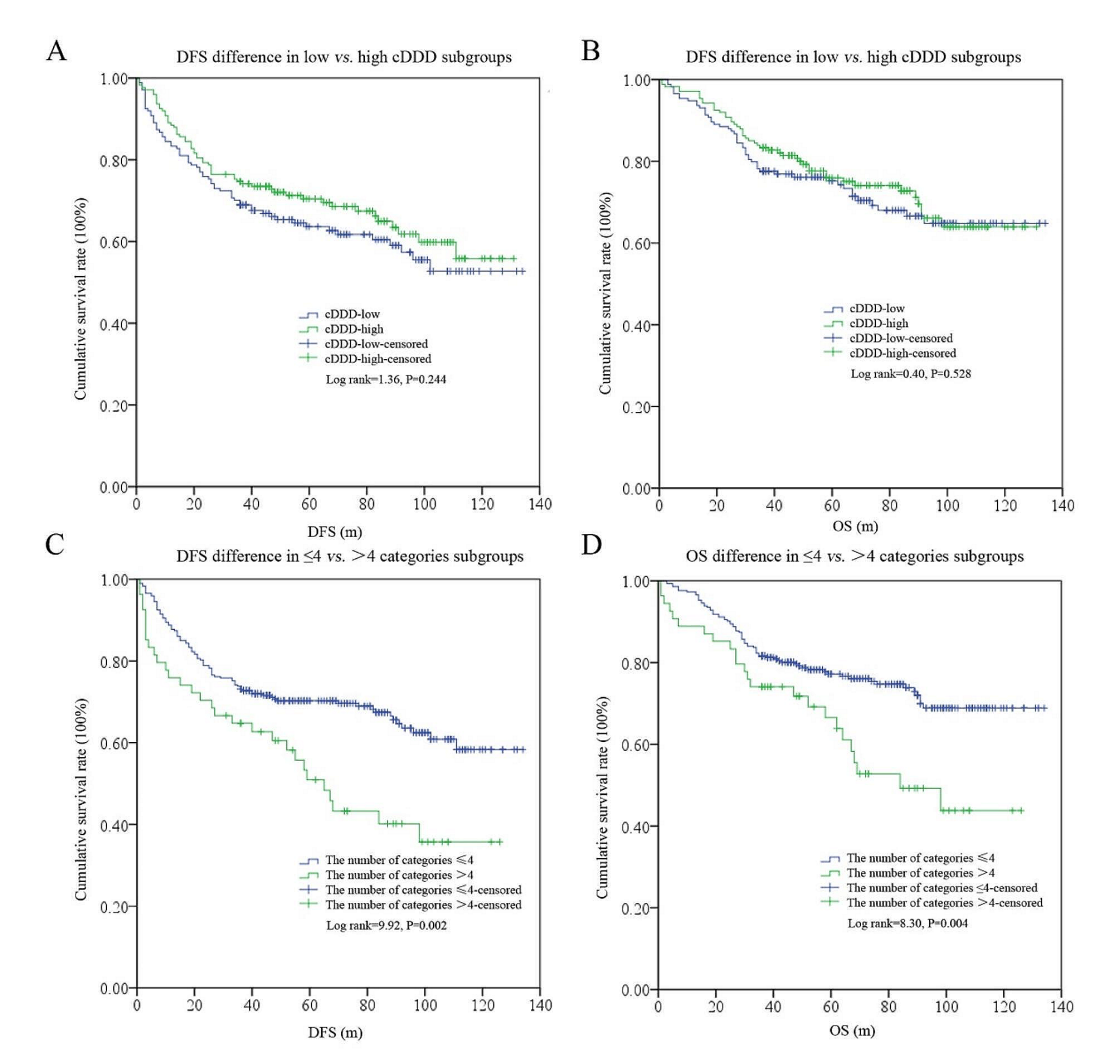
DFS and OS differences among subgroups for different cDDDs (**A**, **B**) and subgroups for the numbers of categories (**C**, **D**)

**Fig. 4:**
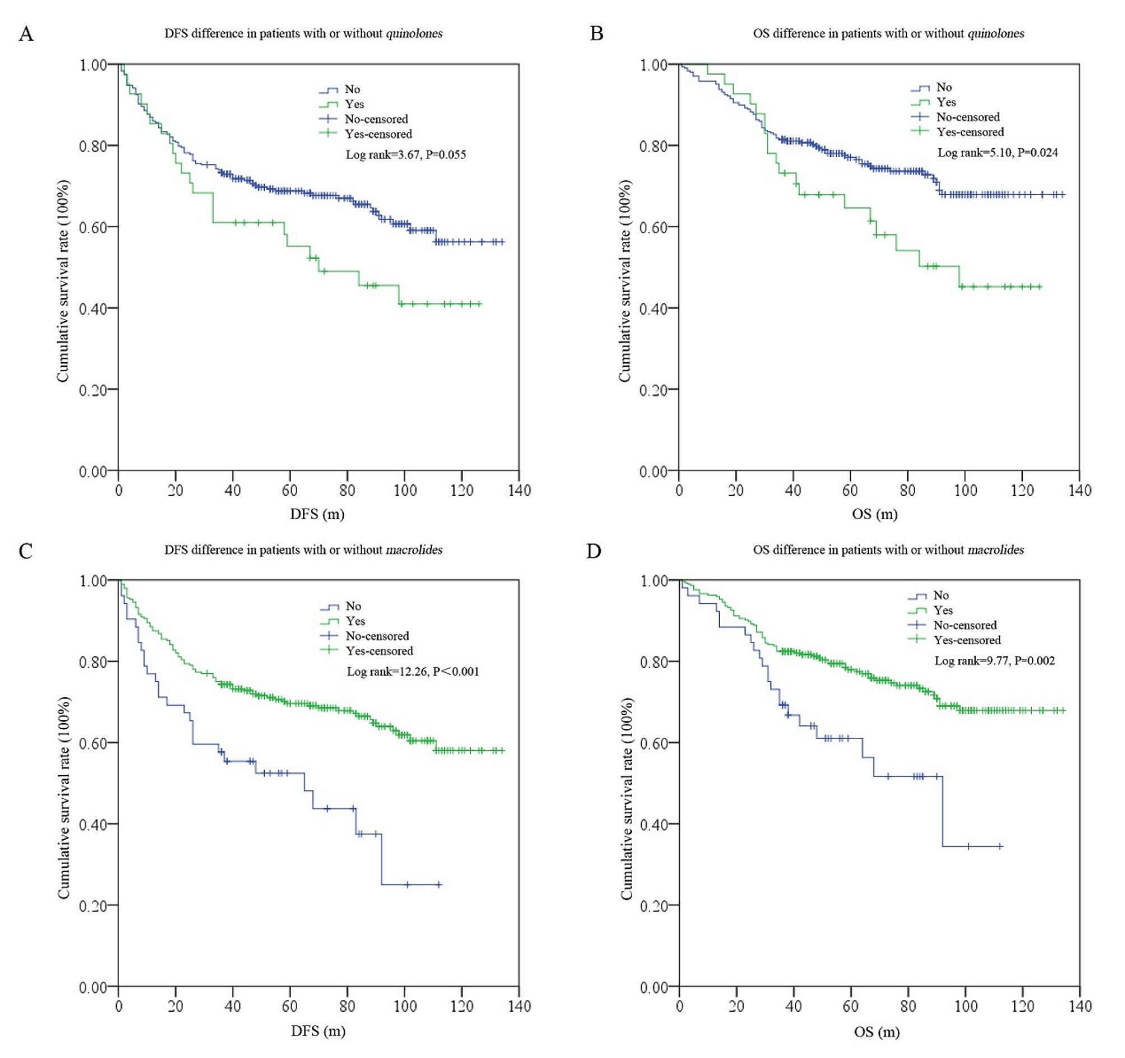
DFS and OS differences among the subgroups for different specific ATB categories (quinolones: **A**, **B**; macrolides: **C**, **D**)

As shown in Fig. 3, no significant differences were found in the low- vs. high-cDDD subgroups for DFS (log rank = 1.36, *P* = 0.244) or OS (log rank = 0.40, *P* = 0.528). However, significant differences were detected in ≤ 4 categories vs. >4 categories subgroups for DFS (log rank = 9.92, *P* = 0.002) and OS (log rank = 8.30, *P* = 0.004). In addition, we further tested differences in survival among patients treated with or without *quinolones* or *macrolides* (differences among patients treated with or without*β-lactams* and *nitroimidazoles* were not detected due to the limited sample size for patients treated without these ATBs). The results indicated that the use of *quinolones* negatively affected the survival (DFS: log rank = 3.67, *P* = 0.055; OS: log rank = 5.10, *P* = 0.024); while the use of *macrolides* improved the survival (DFS: log rank = 12.26, *P*<0.001; OS: log rank = 9.77, *P* = 0.002) (Fig. 4).

### Risk factors for DFS or OS validated by Cox proportional hazards models

As shown in Table 2, resection, obstruction, deposits, T stage, N stage TNM stage, and the number of categories were found to be common risk factors for DFS and OS. These factors were subsequently subjected to multivariate tests, and the results indicated that the number of categories was an independent risk factor for DFS (HR = 2.05, 95%CI: 1.35–3.11, *P* = 0.001) and OS (HR = 1.82, 95%CI: 1.14–2.90, *P* = 0.012) (Table 3).

**Table 2:** Univariate analyses for the risk factor for DFS and OS using the Cox proportional hazards model

	DFS			OS		
	HR	95%CI	*P*	HR	95%CI	*P*
**Age (y)**						
≤ 50			1			1
>50	1.12	0.73–1.74	0.601	1.47	0.86–2.51	0.158
**Sex**						
Female			1			1
Male	1.19	0.82–1.72	0.366	1.07	0.71–1.62	0.745
**Resection**						
Laparoscope			1			1
Laparotomy	2.65	1.78–3.96	<0.001^*^	3.06	1.97–4.76	<0.001^*^
**Obstruction**						
Yes			1			1
No	0.54	0.35–0.83	0.006^*^	0.45	0.28–0.72	0.001^*^
**Tumor location**						
Right			1			1
Left	1.00	0.67–1.48	0.982	0.84	0.55–1.30	0.444
**Histological differentiation**						
Well + moderate			1			1
Poor	1.20	0.70–2.06	0.507	1.05	0.60–1.85	0.864
**Deposits**						
Yes			1			1
No	0.27	0.18–0.40	<0.001^*^	0.24	0.15–0.37	<0.001^*^
**T stages**						
T_1_ + T_2_			1			1
T_3+_T_4_	3.01	1.70–5.35	<0.001^*^	3.28	1.65–6.51	0.001^*^
**N stages**						
N_0_			1			1
N_1+_N_2_	2.83	1.97–4.07	<0.001^*^	2.54	1.69–3.82	<0.001^*^
**TNM stage**						
I			1			1
II	1.86	0.90–3.85	0.096	1.94	0.85–4.41	0.116
III	4.50	2.26–8.97	<0.001^*^	4.18	1.91–9.14	<0.001^*^
**cDDD**						
low			1			1
high	0.81	0.57–1.15	0.247	0.53	0.59–1.31	0.530
**The number of categories**						
≤ 4			1			1
>4	1.91	1.27–2.87	0.002^*^	1.94	1.22–3.08	0.005^*^

*with significant statistical difference

DFS: disease free survival; OS: overall survival; cDDD: cumulative defined daily dose; TNM: tumor-node-metastasis; HR: harzard ratio; CI: confidence interval

**Table 3:** Multivariate analyses for the risk factor for DFS and OS using the Cox proportional hazards model

	DFS			OS		
	HR	95%CI	*P*	HR	95%CI	*P*
**Resection**						
Laparoscope			1			1
Laparotomy	1.88	1.25–2.86	0.003^*^	2.16	1.36–3.43	0.001^*^
**Deposits**						
Yes			1			1
No	0.44	0.28–0.69	<0.001^*^	0.30	0.19–0.47	<0.001^*^
**T stages**						
T_1_ + T_2_			1			1
T_3+_T_4_	2.12	1.17–3.82	0.012^*^	2.53	1.26–5.08	0.009^*^
**N stages**						
N_0_			1			
N_1+_N_2_	1.93	1.28–2.92	0.002^*^			
**The number of categories**						
≤ 4			1			1
>4	2.05	1.35–3.11	0.001^*^	1.82	1.14–2.90	0.012^*^

*with significant statistical difference

DFS: disease free survival; OS: overall survival; HR: harzard ratio; CI: confidence interval

## Discussion

In this study, although the cDDD of ATBs during surgery in stage I-III CRC patients was not significantly correlated with DFS or OS, patients with multiple categories are found to have poor survival. Specifically, the use of *quinolones* seems harmful, whereas the use of *macrolides* was beneficial for survival. Furthermore, the number of categories was found to be an independent risk factor for both DFS and OS. To the best of our knowledge, this is the first report concerning the prognostic value of ATB use during surgery in CRC patients.

In fact, the impact of ATB use on outcomes in cancer patients has been increasingly reported in recent years, particularly in patients who received immunotherapies. Previously, the majority of the studies indicated a negative impact of ATB use in patient survival [10, 11, 21–23]; however, in these studies, the “use” of ATBs was defined only as the use of ATBs before, during or after therapy [24, 25], less consideration was given to the duration or course of ATB use (our study considered this as the cDDD), or ATB category (also referred to as class, species or type in various studies). These problems were further addressed in later studies. For example, Tinsley et al. studied 291 advanced cancer patients and reported that patients who received multiple courses (> 7 days) of ATBs or prolonged ATB treatment had worse PFS and OS than did those without ATBs or a single course of ATBs [17]. In contrast, Cortellini et al. enrolled 302 stage IV NSCLC patients and found no association between the ATB duration (≥ 7 days vs. <7 days) and PFS or OS [26]. In addition, Geum et al. reported that broad-spectrum ATBs, such as *piperacillin*/*tazobactam*, impaired the survival of NSCLC patients who received nivolumab treatment [18]. Similarly, Qiu et al. reported that *quinolones* were less likely to negatively affect patient outcome but that *β-lactams* (*penicillins*, not *carbapenems* or *cephalosporins*) significantly correlated with poor PFS and OS [27]. Interestingly, patients had the worst PFS and OS when *β-lactams* and *quinolones* were used in combination [27], which suggested a synergistic effect between different ATBs. In addition to these patients who received immunotherapies, some studies have indicated that ATB use attenuated the effect of targeted therapy or chemotherapy. For example, Liu et al. suggested that ATB use with targeted therapies led to an inferior PFS; however, they found no differences among patients with different durations (≥ 10 d vs. <10 d) or types (> 1 vs. =1) [12]. Tinsley et al. also concluded that ATB use with targeted therapies correlated not only with poor PFS, but also with poor OS [28]. With regard to CRC, Lu et al. reported a potential correlation between increased mortality and ATBs in patients who received bevacizumab [14]. Abdel-Rahman et al. reported a negative association of PFS and OS with the ATB use before (but not following) the initiation of 5-Fu-based chemotherapies [15]. However, Imai et al. suggested that ATB use could improve the efficacy of oxaliplatin-based rather than irinotecan-based regimens [16]. In our study, we also found that the cDDD of the ATBs (equal to multiple courses, duration or prolonged treatment) during surgery was less likely to play a role in survival; however, we found that the multiple categories could impair DFS and OS, which was partially in line with previous studies in advanced or metastatic malignancies [12, 18, 26, 27].

In recent years, the key role of circulating tumor cells (CTCs) in cancer recurrence, metastasis and treatment failure has been increasingly validated. In CRC, these cells are detectable in up to 78% of stage I-III patients after curative resection [29, 30] and more importantly, some of them display the characteristics of cancer stem cells (CSCs) [31, 32], which are highly resistant to conventional treatment strategies [33, 34]. Interestingly, ATB use was found to play a complex role in cancer development through multiple mechanisms, with the regulation of the gut microbiota (GM) being the most important. Previously, a great number of evidence indicated that GM played an important role in CRC development [35–37] and some species can even manipulate treatment efficacy and toxicity [36, 38, 39]. Some studies have reported obvious dysbiosis of the GM in CRC patients who underwent radical resection [40, 41], and ATB use, particularly of broad‑spectrum ATBs, can greatly disturb these microorganisms [42–44]. More importantly, some reports have suggested that dysbiosis of the GM promotes liver metastasis by remodeling the immune niche or microenvironment in CRC [45, 46]. Based on these facts, it was understandable that the use of ATBs (such as *quinolones*) during surgery correlated with poor survival in our study. However, it was also notable that some ATBs can directly regulate cancer cells and may have a positive anticancer effect. For example, *levofloxacin* was found to contribute to the inhibition of cell proliferation and the induction of apoptosis in lung cancer by regulating mitochondrial dysfunction and oxidative damage [47]. *Moxifloxacin* (another *quinolone*) can contribute to S-phase arrest and the induction of apoptosis by cisplatin in pancreatic cancer through ERK activation [48], inhibit tumor growth or promote apoptosis in breast cancer by interacting with the Mcl-1 and MITF proteins [49]. Nonetheless, these results have not been extensively validated in CRC, particularly in patients with CTCs after surgery. Based on our results, we speculate that the positive role of the above ATBs in preventing cancer could be cancelled out by their disturbance of the GM in CRC [47–49]; however, further studies are needed to address these questions in the future.

Previously, many studies have established the value of prophylactic ATB administration which aims to decrease postoperative infections in CRC patients during surgery with different bowel preparations [5, 7, 8, 50]. In our study, it was further validated that such prophylactic therapy was safe in these patients; however, since patients with multiple categories are likely to have inferior survival, specific caution should be taken when selecting ATBs. In addition, we found that patients treated with *quinolones* or without *macrolides* are likely to have inferior survival. Although these results are partly in line with those of a previous study [27], it should be noted that the number of patients treated with *quinolones* (*n* = 41) or without *macrolides* (*n* = 52) was limited. Furthermore, it was not exclusive the survival of these patients was not exclusively due to their treatment being with *quinolones* or without *macrolides* since the majority of the patients also received *β-lactams* and *nitroimidazoles*, so the putative mutual effect in a previous report [27] cannot be confirmed in our study.

Our work also presented several limitations: first, it was performed retrospectively in single local hospital with a relatively small sample; in particular, the number of patients with or without a specific category was limited, and potential bias cannot be ignored; and second, the molecular information concerning deficient or proficient mismatch repair was absent, as previous studies indicated that the GM could be distinct in these tumors [51]; thus, the impact of ATBs on survival in these patients could also be different. Nonetheless, prospective randomized controlled trials can be conducted to validate our results in the future.

## Conclusion

Overall, our study indicated that the cDDD of ATBs during radical resection in stage I-III CRC patients has no correlation with patient outcome; however, patients in multiple categories are likely to have inferior survival.

## Data Availability

No datasets were generated or analysed during the current study.
